# Fragment-based covalent ligand discovery

**DOI:** 10.1039/d0cb00222d

**Published:** 2021-02-09

**Authors:** Wenchao Lu, Milka Kostic, Tinghu Zhang, Jianwei Che, Matthew P. Patricelli, Lyn H. Jones, Edward T. Chouchani, Nathanael S. Gray

**Affiliations:** Department of Cancer Biology, Dana-Farber Cancer Institute Boston MA 02215 USA nathanael_gray@dfci.harvard.edu; Department of Biological Chemistry and Molecular Pharmacology, Harvard Medical School Boston MA 02215 USA; Center for Protein Degradation, Dana-Farber Cancer Institute Boston MA 02215 USA; Vividion Therapeutics La Jolla CA 92121 USA; Department of Cell Biology, Harvard Medical School Boston MA 02215 USA

## Abstract

Targeted covalent inhibitors have regained widespread attention in drug discovery and have emerged as powerful tools for basic biomedical research. Fueled by considerable improvements in mass spectrometry sensitivity and sample processing, chemoproteomic strategies have revealed thousands of proteins that can be covalently modified by reactive small molecules. Fragment-based drug discovery, which has traditionally been used in a target-centric fashion, is now being deployed on a proteome-wide scale thereby expanding its utility to both the discovery of novel covalent ligands and their cognate protein targets. This powerful approach is allowing ‘high-throughput’ serendipitous discovery of cryptic pockets leading to the identification of pharmacological modulators of proteins previously viewed as “undruggable”. The reactive fragment toolkit has been enabled by recent advances in the development of new chemistries that target residues other than cysteine including lysine and tyrosine. Here, we review the emerging area of covalent fragment-based ligand discovery, which integrates the benefits of covalent targeting and fragment-based medicinal chemistry. We discuss how the two strategies synergize to facilitate the efficient discovery of new pharmacological modulators of established and new therapeutic target proteins.

## Abstract 2

Covalent fragment-based ligand discovery greatly facilitates the discovery of useful fragments for drug discovery and helps unveil chemical-tractable biological targets in native biological systems.

## Graphical abstract

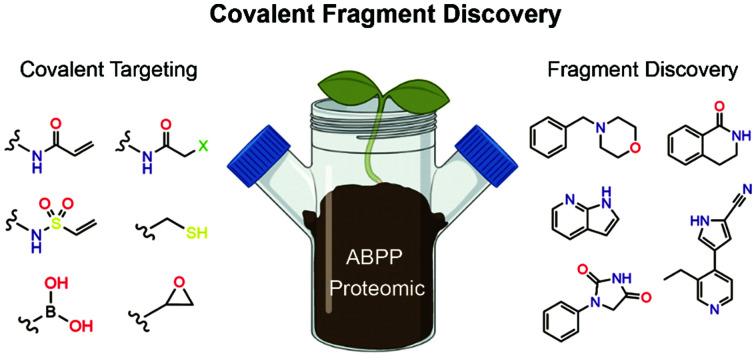

## Introduction

Inspired by recent approval of covalent kinase inhibitors (TKIs) for cancer treatment including inhibitors targeting EGFR: afatinib (Gilotrif) and osimertinib (Tagrisso) or BTK: acalabrutinib (Calquence) and ibrutinib (Imbruvica), the development of covalent probes and drugs has undergone a renaissance and now attracts intense interest from both industry and academia.^1–4^ Unlike noncovalent small molecules that target conserved substrate and/or allosteric binding site transiently, covalent inhibitors often exhibit differentiated pharmacology in terms of potency, selectivity, pharmacokinetics and pharmacodynamics as a consequence of their ability to form irreversible, covalent bonds with their target proteins.^5,6^ Despite these advantages, many viewed covalent inhibitors skeptically due to their ability to result in protein adducts capable of triggering idiosyncratic immune responses and allergic/hypersensitivity reactions.^7,8^ Historically the discovery of covalent drugs was often serendipitous based on natural products such as the beta-lactam class of antibiotics exemplified by the discovery of penicillin or based on rational structure-based design from non-covalent inhibitors as exemplified by the development of covalent kinase inhibitors targeting EGFR or BTK. However, there has been a resurgence of interest in covalent inhibitors that has resulted in a host of new approaches for discovering and developing covalent inhibitors, resulting in 7 covalent drugs (from the total of 161 small molecule drugs) approved by the FDA from 2015 to 2019.^9^

Rational covalent inhibitor design usually starts with a known noncovalent binder and explores strategies to incorporate an appropriate electrophilic warhead to achieve desired target selectivity and efficacy (“binder-first” approaches).^2,3,10^ While this approach has been successful, it is limited to targets that: (1) have existing ligands amenable to further derivatization with a reactive warhead; and (2) include a suitably reactive residue within or near the noncovalent ligand binding site.^11^ Moreover, although recent chemoproteomic studies have identified additional protein targets that feature at least one reactive residue suitable for covalent strategies, many of these sites are located within cryptic ligand pockets, for which reversible ligand discovery remains difficult.

Fragment-based drug discovery (FBDD) has been widely applied as an alternative to large chemical library screening for ligand discovery, especially in the context of intractable biological targets.^12–14^ The FBDD approach for discovery of noncovalent binders involves assembling a library of low molecular weight ligands (typical MW of less than 300 Da) called fragments, which are screened against a purified target of interest to identify low affinity binders with high ligand efficiency (LE). The fragments are subsequently elaborated and optimized to yield higher affinity ligands.^15^ Typically, FBDD screening can yield a lead from a limited size library (few thousand fragments *vs.* hundred thousand and larger libraries typically used for high-throughput screening of drug-like libraries). Another advantage of fragments is their ability to access cryptic sites beyond substrate pockets, which holds great promise for allosteric drug discovery and discovery of ligands for targets that lack well-defined binding pockets.^16^ However, one of the liabilities of fragment-based strategies is the low intrinsic binding affinity of the fragments, making these interactions sometimes difficult to detect. Additionally, fragment optimization into higher affinity, selective ligands is difficult, and places significant emphasis on the need for structural biology information. Lastly, validation of fragment binding and on-target mechanism of action *via* genetic methods is currently not possible.

**Fig. 1:**
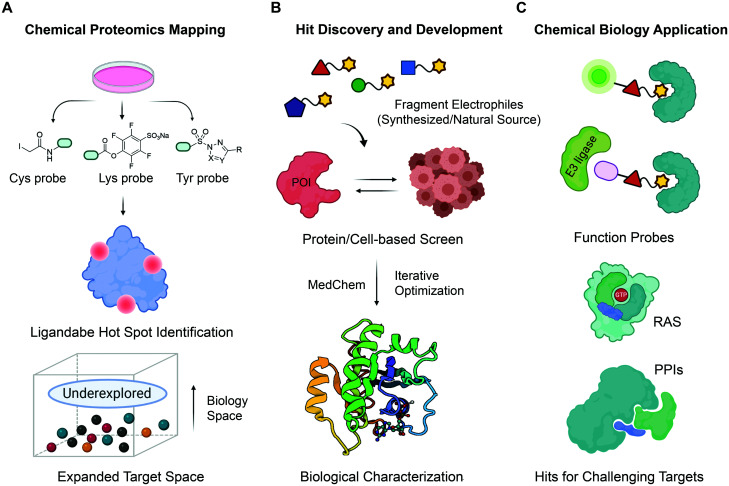
The roadmap for fragment-based covalent ligand discovery. (A) State-of-the-art chemoproteomic strategies helps to systematically unveil potential ligandable sites in disease-associated targets. Cells are treated with reactive fragments (biased towards thiols (Cys), amines (lysine) and phenols (tyrosine)) and then chemoproteomics allows identification of proteins and reaction sites. (B) Fragment-based covalent ligand screening identified covalent fragment hits, which can be evolved into a more potent, selective, biocompatible and drug-like ligands by iterative elaboration and optimization. (C) Fragment-based ligand discovery pipeline holds great promise as an initial ligand-discovery approach that can be elaborated to make bivalent molecules that can recruit other enzymes including E3s for PROTACs *etc*.

One way to mitigate challenges related to both FBDD and the “binder-first” approaches for covalent ligand discovery is to use covalent fragments in FBDD. Covalent fragments have a potential to achieve excellent target engagement *via* covalent bond formation, and selectivity by reacting with distinct target residues. Additionally, due to covalent nature of their interaction with the target, target engagement and on-target mechanism of action can be unequivocally established using mass spectrometry and confirmed through target residue mutagenesis. Besides, optimization of covalent fragments could be more straightforward since their binding mode does not change easily during fragment merging/growing. These benefits of covalent fragments in drug discovery has resulted in increased interest in this area. Therefore, this review aims to provide an overview of the recent efforts to discover, develop and validate covalent fragments as a promising strategy for covalent ligand discovery. We will discuss ways to discover new covalent fragments, as well as how covalent fragments are used in chemoproteomics for: (1) mapping proteomic reactivity of the targets; (2) identifying new hotspots for inhibitor/ligand development; and (3) mapping compound selectivity. We will also provide a more comprehensive overview of the recent advances in expanding the repertoire of electrophiles towards protein nucleophiles other than cysteine and highlight some important chemical biology applications of covalent fragments (Fig. 1). We would like to note that several excellent reviews focusing on covalent fragment library design have recently been published,^1,2,17–19^ and we will not comment on this here.

## Strategies for covalent fragment discovery

### Target-based covalent fragment discovery

**Fig. 2:**
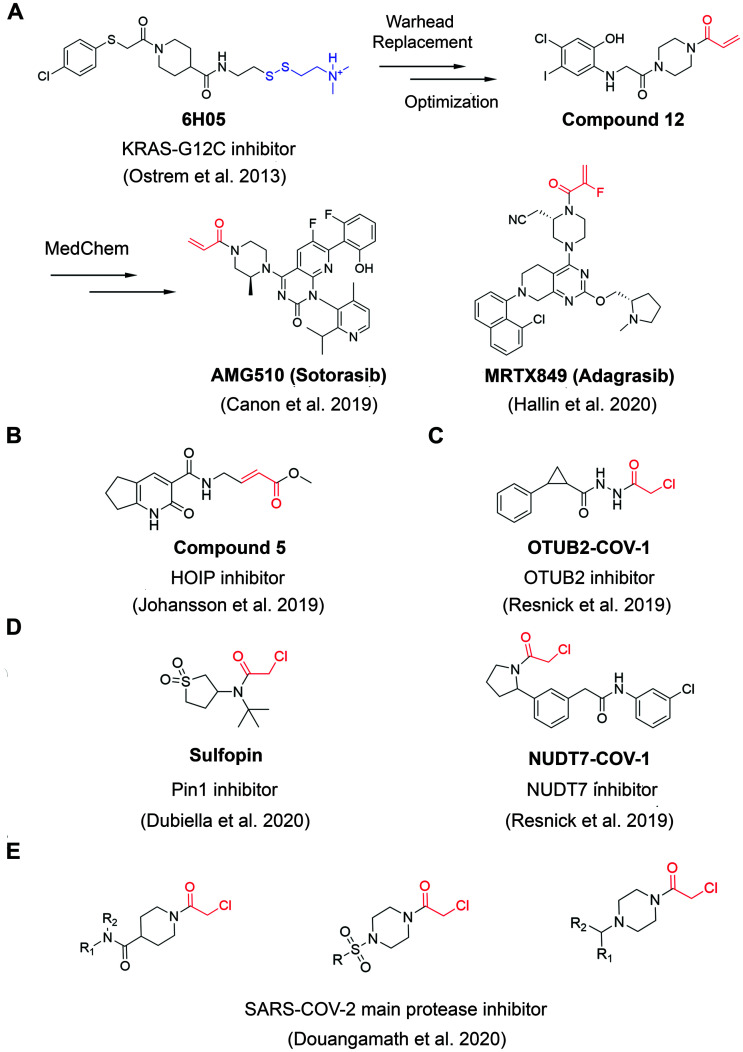
The structures of representative well-characterized electrophilic fragments identified from target-based screening strategies in recent years. (A) KRAS-G12C allele-specific covalent fragment (6H05) identified from tethering screen, which was further elaborated to compound 12.^31^ This inspired numerous groups to develop further optimized inhibitors, within which AMG510^33^ and MRTX849^36^ successfully entered clinical trials. (B) Compound 5 targets the active cysteine (C885) of HOIP.^37^ (C) OTUB2-COV-1 targets the active cysteine (C51) of OTUB2 and NUDT7-COV-1 target C73 of NUDT7.^38^ (D) Sulfopin targets the active cysteine of Pin1 (C113).^39^ (E) Representative covalent fragment scaffolds target the active cysteine (C145) of SARS-COV-2 main protease (M^pro^).^40^

A widely applied covalent fragment screening strategy is called site-directed disulfide tethering, also known as disulfide trapping.^19–21^ The tethering compound usually contains a cysteamine group to increase compound solubility and a disulfide group to capture naturally occurring or proteins with a cysteine introduced by site-directed mutagenesis through rapid thiol exchange under reducing conditions. The disulfide part of hit compounds is typically replaced by a more stable moiety or other cysteine-directing warheads like acrylamides in follow-up studies in order to improve drug-like properties. Given its convenience and versatility, the covalent tethering method has been widely adopted in drug discovery programs for different protein classes including enzymes, G protein coupled receptors (GPCRs) and protein–protein interactions (PPIs).^22–28^ Perhaps the most successful application of disulfide tethering was the identification of fragments targeting allele-specific K-Ras^G12C^ oncoprotein.^29^ Genetic alterations of Ras family proteins are the most prevalent oncogenic mutations in cancer cells. However, Ras oncoproteins has been considered to be undruggable due to the picomolar binding affinity for GTP substrate.^30^ In 2013, Ostrem *et al.* screened a set of 480 tethering fragments against K-Ras^G12C^*in vitro* by intact protein mass spectrometry.^31^ The screen led to the identification of fragment 6H05 that could bind to the allosteric site of K-Ras^G12C^ protein, in a ligand-induced pocket named the switch-II pocket with great selectivity over wildtype K-Ras due to the unique G12C residue. Iterative chemistry optimization, including replacement of the disulfide group with more stable carbon-based electrophiles like acrylamides, resulted in promising lead compounds such as compound 12. Importantly, these studies served as a proof-of-ligandability concept for K-Ras^G12C^, which inspired numerous groups to develop further optimized inhibitors,^32–36^ including AMG-510 (proposed INN sotorasib), which is the first drug candidate entering clinical stage for the treatment of advanced/metastatic solid tumors with KRAS^G12C^ mutation (NCT03600883, NCT04380753), and MRTX849 (adagrasib) (Fig. 2A). These studies have contributed significantly to the increased interests in using covalent fragments for drug discovery and development.

**Fig. 3:**
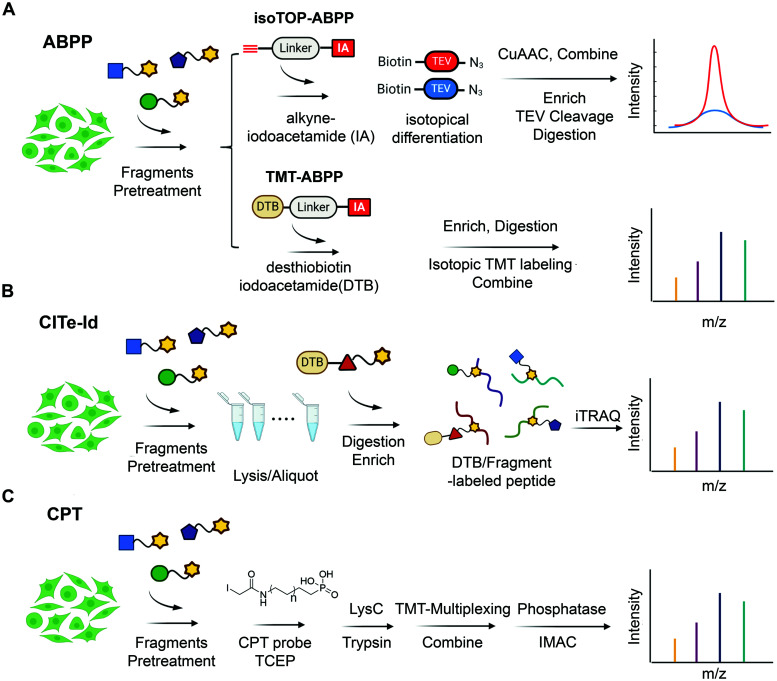
State-of-the-art chemoproteomic approaches for reimaging druggable proteome and selectivity profiling of covalent fragments. (A) Activity-based protein profiling (IsoTOP-ABPP and TMT-ABPP) using isotope-labeled probes or isotopic TMT labeling agents for multiplex quantitative chemoproteomics.^39,54–57^ (B) Covalent Inhibitor Target-site Identification (CITe-Id), another complementary chemoproteomic platform to understand the proteome-wide on/off-target effect in covalent fragment development program, in which desthiobiotinylated covalent inhibitor is used in lieu of non-selective iodoacetamide probe to directly monitor target engagement.^58^ (C) Cysteine-Reactive Phosphate Tags (CPTs) developed recently can be applied as a chemoproteomic using phosphate-tagged iodoacetamide for global cysteine profiling with high coverage of the cysteine proteome.^59^

Although covalent tethering has been successfully used for fragment discovery, disulfide-containing fragment libraries are not generally available from commercial vendors. Additionally, considering the metabolic liability of disulfide group and complex redox environment *in vivo*, this strategy requires significant chemistry efforts post hit identification to replace the disulfide group with more suitable warheads.^41^ Therefore, additional electrophilic fragment screening strategies have been developed in recent years.^42–47^ For example, Johansson *et al.* recently designed and synthesized a 104-fragment library of α,β-unsaturated methyl ester warheads, which have been demonstrated to exhibit narrower reactivity profile compared with other commonly used electrophiles, such as acrylamides.^37^ The fragments were screened against the E3 ligase HOIP RBR domain in 22 pools by LC-MS assays. Among the hits, compound 5 (Fig. 2B) was identified as a covalent hit that targets catalytic Cys885 with *k*_inact_/*K*_I_ value of 0.97 ± 0.01 M^−1^ s^−1^ (*k*_inact_/*K*_I_ value is an important measure of covalent inhibitor performance, and we refer readers interested in learning more to a recent publication on this topic^48,49^). The compounds showed great selectivity against a panel of RBR E3 ligases, HECT E3 ligases, E1/E2 enzymes and deubiquitinating enzymes. Further activity-based protein profiling (ABPP; Fig. 3A) study using a slightly more potent derivative demonstrated the on-target effect in cells. This work shows the potential of fragment-based covalent ligand screening for first-in-class drug discovery against targets like E3 ligases, which have traditionally been challenging. Moreover, given the importance of novel E3 ligase ligands for targeted degrader development,^50^ strategies like this one could substantially expand the toolbox of E3 ubiquitin ligases that can be exploited in this context.

Another example aimed at finding covalent fragments for difficult-to-drug cysteine-containing proteins, used a larger fragment library of 993 covalent building blocks containing acrylamides or chloroacetamides to screen against 10 proteins, including bovine serum albumin (BSA) as a negative control.^38^ Among those targets, the intact protein MS screen led to discovery of 37 strictly nonpromiscuous hits with >50% labeling efficiency. Streamlined high throughput crystallographic studies resulted in the successful determination of 15 inhibitor-protein complex structures. Further fragment analogue evaluation yielded the best inhibitor OTUB2-COV-1 (Fig. 2C) with the *k*_inact_/*K*_I_ value of 3.75 M^−1^ s^−1^. IsoTOP-ABPP demonstrated that OTUB2-COV-1 (10 μM compound, 2 h incubation) labeled <1% probe-accessible cysteines (Heavy/Light ratio >4) in HEK293T cells suggesting promising selectivity profile suitable for further development. Additionally, screening against pyrophosphohydrolase NUDT7 yielded 20 strictly nonpromiscuous hits with a total hit rate of 2%, which did not label two or more proteins by more than 30%. Fragment merging with previously identified noncovalent hit led to the discovery of NUDT7-COV-1 (Fig. 2C) with the *k*_inact_/*K*_I_ value of 757 M^−1^ s^−1^. Cellular target engagement was further validated by cellular thermal shift assay, providing additional support that these types of screens of limited covalent fragment libraries can yield high quality leads for development of selective ligands for challenging targets.

Very recently, screening the same library of 993 fragments with acrylamides or chloroacetamides warheads followed with rational chemistry optimization, led to the discovery of a potent and selective covalent Pin1 inhibitor, called Sulfopin with the *k*_inact_/*K*_I_ value of 84 M^−1^ s^−1^ (Fig. 2D).^39^ Pin1, a peptidyl-prolyl *cis*–*trans* isomerase, has been a challenging target and Sulfopin therefore represents an important addition to the currently available Pin1 ligands. To confirm the mode of binding, crystallographic studies clearly demonstrated that Sulfopin binds to Pin1 covalently to Cys113. Despite the relatively small size of the fragment and the typically more reactive chloroacetamide electrophile, Sulfopin exhibited impressive selectivity in chemoproteomic experiments and significantly retarded neuroblastoma initiation in zebrafish tumor model.

Using a compound library with over 1250 fragments, Douangamath *et al.* conducted a combined mass spectrometry and high throughput crystallographic fragment screen against SARS-CoV-2 main protease (M^pro^), which is an essential target for viral replication. This screen finally yielded 48 high-value covalent fragments co-crystallized with M^pro^ featuring a variety of scaffolds, which offered unprecedented structural resources for the follow-up structure-based anti-viral drug discovery^40^ (Fig. 2E).

Besides the hit identification, covalent fragment-based approaches are also suitable for mapping the protein hot-spots. Very recently, Petri *et al.* described a library of covalent fragments with identical scaffold but chemically diverse electrophilic warheads. They screened this collection against a panel of kinases with at least one targetable cysteine (BTK, ERK2, RSK2 and MAP2K6), and used JAK3 and MELK for further validation studies. This study demonstrated that covalent fragments can be used to map cysteine tractability across a wide range of targets^51,52^ Collectively, these recent examples illustrate the robustness and efficiency of electrophilic fragment screening, as well as the potential for identifying non-promiscuous covalent hits and evaluating the accessibility and reactivity of targeted cysteines.

### Cell-based covalent fragments discovery

Although target-based electrophilic library screens described above are effective and relatively easy to implement, they require production and isolation of stable recombinant target protein. For many targets of interest, this is either not feasible on a scale needed for these experiments or impossible given intrinsic instability of the target. Additionally, in many cases selectivity against isolated protein does not directly translate into global chemoselectivity in cellular environment. To address this, several cell-based screens of electrophilic fragment libraries using state-of-art chemoproteomic strategies have recently been conducted (Fig. 3).^53^

**Fig. 4:**
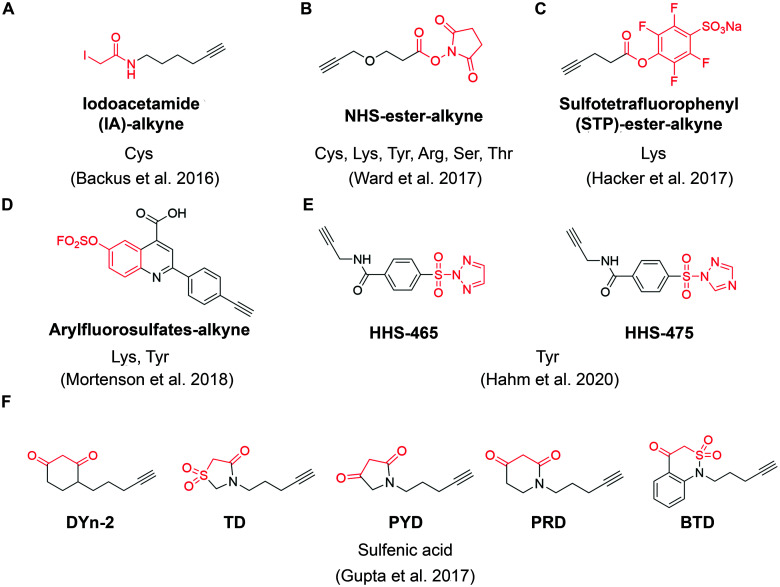
The structures of representative ABPP probes applied in state-of-the-art chemoproteomic strategies. (A) Iodoacetamide-alkyne is a widely applied cysteine-reactive ABPP probes.^54^ (B) NHS-ester-alkyne is a versatile covalent ABPP probe for protein nucleophiles (cysteines, lysines, tyrosines, serines, threonines, and arginines).^65^ (C) STP-alkyne is an amine-reactive covalent ABPP probe.^67^ (D) Latent electrophiles as exemplified by SuFEx-based arylfluorosulfates for lysine/tyrosine targeting.^72^ (E) HHS-465 and HHS-475 are SuTEx-chemistry based ABPP probes for tyrosine profiling.^74^ (F) Carbon nucleophiles help to explore ligandability of sulfenylated (oxidized) cysteines.^75^

In 2016, Cravatt *et al.* used cysteine-reactive ABPP probes (Fig. 4A) to conduct proteome-wide screening of 56 cysteine-directing fragments containing chloroacetamide or acrylamide electrophiles. The screen demonstrated that ∼20% of quantified proteins harbored ligandable cysteine including transcription factors, adaptor and scaffold proteins. One of the hits identified both *in vitro* and *in situ* is pro-caspase 8/10 fragment that could selectively modify pro-caspase 8 and not the active form, caspase 8. Further chemistry efforts led to the development of selective caspase 8 inhibitor, thus demonstrating that covalent fragment hits identified in this manner are readily optimizable.^54^

Following on this work, Cravatt and colleagues have expanded the scope of their studies further. For example, by using broadly cysteine-reactive fragments as “scout fragments”,^54^ they examined proteomic cysteine ligandability in *KEAP1*-mutant and *KEAP1*-wild-type (WT) human non-small cell lung cancer (NSCLC) cell lines. Among more than 1000 cysteine sites that exhibited sensitivity to “scout fragments”, the authors detected orphan nuclear hormone receptor NR0B1 (modified on Cys247). Follow up analysis demonstrated that NR0B1 expression was associated with KEAP1 mutational status, and that covalent inhibitor targeting Cys247 inhibited anchorage-independent NSCLC cell growth.^60^ Very recently, they further investigated ligandability of cysteines in primary human T cells using a similar electrophilic “scout fragment” strategy.^61^ This work generated a view of cell-state dependent ligandable cysteines in human T cells, and the study further suggested that immunomodulatory compounds can be obtained rapidly *via* elaboration of electrophilic compounds. Together, these recent results demonstrate the great potential of phenotypic electrophilic fragment screening for identifying lead fragments for further optimization as well as additional ligandable targets suitable for covalent inhibitor development.

Although covalent targeting cysteine has achieved great success, cysteine is one of the least abundant amino acids with ∼2% occurrence in human proteome, limiting the number of potentially targetable sites.^62,63^ Furthermore, cancer cells are able to acquire resistance through simple oxidation/mutation of the target cysteine, thus making the drug ineffective.^64^ Therefore, in addition to cysteine side chains, lysines, threonines, serines, tyrosines and potentially other side chains may represent an opportunity for screening covalent fragments.

For example, Ward *et al.* deployed *N*-hydroxysuccinimide-ester (NHS-esters) (Fig. 4B) as versatile ABPP probes to profile ligandable protein nucleophiles (primarily lysines) in mouse liver proteomes.^65^ This work led to the discovery of more than 3000 potential hotspots within the proteome that could potentially be explored for development of covalent ligands. Based on the competitive platform, the authors then screened NHS-ester containing fragments leading to the identification of selective covalent probes for dihydropyrimidine dehydrogenase (DPYD), aldehyde dehydrogenase (ALDH2), and glutathione S transferase theta 1 (GSTT1). Although the authors documented potential targeting of residues beyond cysteines and lysines (such as serines, threonines, tyrosines and arginines), it is important to note that NHS-ester reactivity strongly favors lysines, thus the potential for this group to be used for chemical probe development towards residues other than lysine is unclear. Nonetheless, the work further supports a view that side chains beyond cysteine can be used in this context, which would certainly expand the potential of covalent fragment based approaches.^66^

In another example, a lysine-specific, amine-reactive sulfotetrafluorophenyl (STP) alkyne probe (Fig. 4C) was used to screen ∼30 lysine-reactive electrophilic fragments.^67^ The study identified a number of ligandable lysine residues, as well as resulted in the identification of lysine-directed covalent ligands targeting enzymatic sites (PNPO-K100, NUDT2-K89), allosteric pockets (PFKP-K688) and protein–protein interactions (PPIs) (SIN3A-K115). Very recently, Wolter *et al.* described a concept of imine tethering, which uses fragments featuring aldehyde groups to react with lysine side chains.^68^ The authors applied this strategy for discovery of PPI stabilizers (“molecular glues”) of 14-3-3/NF-κB interaction. Targeting of an interface lysine with unique (low) p*K*_a_ (Lys122) was confirmed using X-ray crystallography, and further optimization of the initial fragment lead resulted in two new aldehyde-containing stabilizers of this PPI, as validated in biochemical assays. Overall, this strategy may open additional opportunities for discovery of PPI stabilizers (“molecular glue” compounds^69^) of other complexes.

Beyond lysine targeting, the development of SuFEx (Sulfur Fluoride Exchange) chemistry enables the covalent fragment screening against tyrosine in human proteome.^70,71^ In 2018, Kelly group employed “inverse drug discovery” strategy using latent electrophiles as exemplified by aryl fluorosulfates fragments (Fig. 4D), to survey the ligandable sites in HEK293T cells.^72^ This led to the identification and validation of covalent ligands for 11 important proteins. Very recently, they developed 16 structurally diverse sulfuramidimidoyl fluoride (SAF) functionalized probes to expand the SuFEx toolbox for inverse drug discovery in proteome. 72% of the protein targets identified by SAFs have not been previously identified in previous SuFEx-based chemoproteomics.^73^

Hsu *et al.* have recently adapted sulfur-triazole exchange (SuTEx) chemistry (Fig. 4E) for fragment-based ligand discovery of Tyr-directed binders.^74^ By fine-tuning the SuTEx reactivity, they identified more than 10 000 SuTEx-reactive tyrosines and phosphotyrosines in human proteome. This suggests that SuTEx chemistry is suitable for developing Tyr-reactive covalent inhibitors as well as covalent fragments.

In addition to electrophilic covalent fragments, the Carroll group has recently reported a novel class of nucleophilic covalent fragments targeted at sulfenylated (oxidized) cysteines.^75^ Partial oxidation of cysteine to the sulfenic acid is a physiological process that has been exploited to develop chemical probes that only recognize a particular oxidation state of a protein. These sulfenic acid-reactive covalent ABPP probes (Fig. 4F) has resulted in the identification of more than 1280 sulfenylated cysteines in the colon carcinoma cell line RKO, which shed light on the development of covalent fragments therapeutically targeting redox-active cysteines. Overall, in a way similar to covalent inhibitors, covalent fragments directed at different residues are achievable, and their use as chemoproteomic profiling probes in cell-based assays offers a promising strategy for identifying new targetable sites within the proteome.

We would like to note that so far one of the biggest barriers to all of the chemoproteomic approaches is throughput and the current published chemoproteomic methodologies are limited to very small libraries (usually <100 molecules). Another barrier is the stochastic nature on MS data acquisition that makes it difficult to interpret structure–activity relationship (SAR) trends when screening compound derivates collections. The above challenges are due to the fact that most chemoproteomic approaches to date rely on pairwise labeling strategies, which limit both throughput and consistent identification and quantification of target engagement proteome wide. The development of multiplexing-based chemoproteomic methodologies is an important step towards addressing the throughput issues. The most advanced of these technologies makes use of tandem mass tag (TMT) reagents, isobaric amine-reactive molecules that label N termini of peptides and the ε-amino groups of lysines. With both 10plex, and recently developed 16plex varieties, TMT labeling strategies provide an opportunity to greatly increase throughput and reproducible proteome coverage in chemoproteomic workflows.^76^ One additional strategy to overcome these limitations is to employ a gene family approach, where screening is done against a complete (or nearly complete) gene family. One well-stablished example of this strategy is KiNativ™ platform for *in situ* kinase profiling, in which users can obtain complete coverage for target of interest in each run when screening a library, and achieve higher quantitative accuracy.^77^

As with noncovalent FBDD, many covalent fragments that emerge from these efforts remain far from ideal in terms of potency, thus requiring systematic medicinal chemistry optimization. We expect that continued advancement and implementation of chemoproteomic methods in terms of library size and associated throughput and closer collaboration between mass spectrometry professionals and medicinal chemists might reveal many more opportunities in drug discovery.

### Photoactivation-assisted fragment discovery

**Fig. 5:**
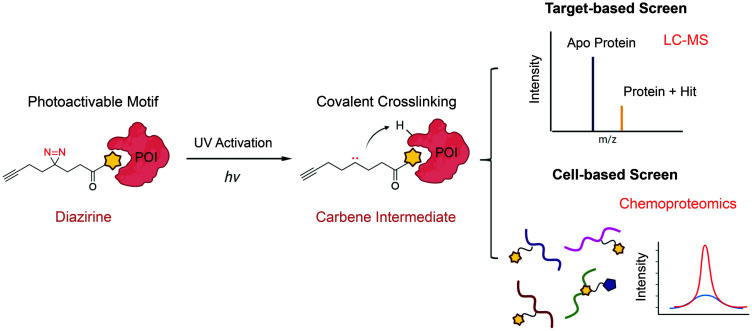
Schematic of photoactivation-assisted fragment discovery. Photoreactive fragments contains a photoactivable diazirine, which could generate the carbene intermediate upon UV activation that can crosslink to proximal protein residues. Photoactivation-assisted fragment discovery has broad applicability in both target-based^79^ and cell-based screening^78^ campaigns.

The major limitation of chemoproteomic studies described thus far is that they can only identify targets featuring suitably reactive nucleophilic (primarily cysteine) residues, which may significantly narrow the target space. To circumvent this, several research groups developed fragment screening platforms that use photoactivatable groups, such as diazirine. In this case, a fragment remains inactive until activated by UV irradiation, which in the case of diazirines generates a reactive carbene intermediate that can react with any amino acid side chain or peptide backbone. Therefore, once bound to the protein pocket, the photoreactive fragment could crosslink to adjacent residues, resulting in an adduct that can be identified using MS (Fig. 5). Parker *et al.* used 14 photoactivatable (diazirine group-containing) fragment probes featuring different chemical structures to profile fragment target space in HEK293T cells.^78^ Using quantitative MS-based proteomics, they identified more than 2000 protein targets, including many proteins for which no ligand has previously been reported in DrugBank. Using a similar strategy, researchers from GSK developed a fragment screen platform named PhotoAffinity Bits (PhABit) for rapid fragment hit identification.^79^ The resulting fragment-protein adducts could be easily detected by LC-MS and MS/MS approaches result in the identification of the sites of covalent modification. As proof-of-concept, they established a compound collection of 556 PhABits and screened against a variety of targets including BRD4-BD1, BCL6 and KRas4B^G12D^. This enables rapid identification of several selective hits that have been successfully verified by follow-up biophysical studies.

Overall, one major advantage of using photoactivatable groups in FBDD screens is that the fragment interactions with targets are driven by LE, while photoactivation and subsequent covalent adduct formation overcomes the low *K*_d_ values associated with noncovalent fragments. Therefore, we expect that these types of strategies will continue to play an important role in fragment screening and discovery. However, it should be noted that fine tuning of the photolabeling method is required to ensure specificity and avoid the formation of putative long-lived species, which may lead to diffusion-based, non-specific crosslinking.

## Computer-aided covalent fragment design

In addition to methods described above, computational methodologies and software have been recently tailored towards covalent fragment-based ligand discovery. Designing covalent fragments with proper warhead reactivity is of utmost importance in covalent ligand discovery. In recent years, a variety of computational approaches have been investigated to estimate warhead reactivity based on quantum mechanics (QM) calculation and experimental calculated intrinsic warhead reactivity. Some of the warheads analyzed in this manner include nitrile-carrying compounds,^80^ Michael acceptors,^81^ acrylamides and 2-chloroacetamides.^82^

Besides predicting the reactivity of a warhead, understanding reactivity of protein nucleophiles is also of paramount importance. The p*K*_a_ of a typical lysine side chain is around 10.4, which means under physiological conditions, they are protonated and cannot readily react with electrophiles.^83^ However, the p*K*_a_ of lysine could be perturbed by its environment, thus changing its reactivity towards covalent fragments.^84^ In 2019, Liu *et al.* developed a continuous constant pH molecular dynamics (CpHMD) for lysine nucleophilicities prediction.^85^ This new strategy successfully identified catalytic lysines in 8 human kinases, which hold great promise for the development of lysine-targeting covalent kinase inhibitors. Overall, computational reactivity prediction methodologies could be potentially useful for virtual compound library design as well as for guiding covalent fragment library design in general.

Free energy calculation is considered to be a robust computational approach for binding affinity prediction, and this strategy has been successfully extended to the development of covalent inhibitors. For example, Chatterjee *et al.* established a two-state model for selectivity prediction of reversible covalent inhibitors among protein homologues.^86^ Very recently, Mihalovits *et al.* also extended free energy calculation for the affinity and selectivity evaluation of irreversible binders as exemplified by covalent inhibitors targeting K-Ras ^G12C^ and EGFR.^87^

Another important advancement has been the development of covalent docking approaches.^88–90^ Structure-based virtual screening has been successfully adopted to enrich active compounds from large compound libraries.^91^ In response to growing interest in targeted covalent inhibitors, many computational platforms have integrated covalent docking modules to assist drug discovery campaigns.^88,92–100^ For example, London *et al.* described a method called DOCKovalent for large-scale covalent fragment virtual screening campaigns (http://covalent.docking.org/).^88^ In this method, the receptor nucleophiles were set as rigid while the fragment conformations and possible stereoisomers were exhaustively sampled with constraints on the distance and angle of the covalent bond. The scoring was calculated based on non-covalent interactions to evaluate structural complementarity without direct calculation of covalent bond energy. This method has been successfully applied to target different nucleophiles and discover reversible covalent inhibitors targeting AmpC β-lactamase, RSK2, MSK1 and JAK3 kinases by screening electrophile fragment libraries. Very recently, Tang *et al.* developed an artificial intelligence (AI)-based design strategy namely advanced deep Q-learning network (ADQN) for covalent ligand discovery targeting SARS-CoV-2 3CL^pro^ which is also a nice example of how artificial intelligence make the hunt quickly and efficiently.^101^

In another example, Robert *et al.* generated a covalent 3D pharmacophore collection based on protein–ligand interactions, which was subsequently applied for covalent fragment screen against enteroviral 3C protease.^102^ By screening against a compound collection of approximately 3000 fragments, scaffold hopping and follow-up biological evaluation, they identified phenylthiomethyl ketone-based fragments as 3C protease inhibitors.

Despite these successes, one of the major bottlenecks in this field is that the docking performance varies when it comes to different targets making it challenging to develop the most suitable and applicable scoring function for hit enrichment or accurately rank the hits. Additionally, most current covalent docking tools fail to calculate covalent bond energy, which relies on computationally expensive QM calculations. Proper consideration for covalency, stability, reaction rate, and potential reversibility add layers of complexity to the calculations and it remains to be seen whether those methodologies could be generally applied against a broader range of targets. We believe that based on vastly increasing amount chemoproteomics data, the community efforts will enable the development and validation of new computational methods including modern machine learning algorithms for *in silico* screening of covalent virtual library and rational design of covalent ligand for targets of interest.

## Advancing challenging pharmacological modalities

### Inhibition of protein–protein interactions

Fragment-based ligand and drug discovery approaches have a great potential to drug some of the more challenging targets, such as protein–protein interactions (PPIs).^103^ There are estimated 650,000 PPIs in human proteome.^104^ However, due to the large and flat nature of most PPI interfaces, it remains quite challenging to develop small molecules to target them.^105,106^ Due to the covalent nature, covalent fragments have the ability to bind when the partners are apart, which could avoid the huge loss of potency due to competition with the complex. This could be of special importance when targeting signaling pathways where interactions are regulated and/or transient. Thus, leveraging the covalency may help address shallow pockets and offer additional opportunities for chemical intervention against PPIs by irreversible attachment.

In 2015, by screening covalent fragments, Statsyuk *et al.* identified a covalent inhibitor targeting non-catalytic Cys627 located at the HECT domain of Nedd4-1, an E3 ubiquitin ligase.^45^ The covalent mechanism of action (MOA) was validated by mass spectrometry and crystallographic study. Further chemistry optimization led to the development of more potent inhibitor which could disrupt Nedd4-1:Ub interaction with *k*_inact_/*K*_I_ value of 1.98 M^−1^ s^−1^. In another example, starting from non-covalent fragment hit, researchers from AstraZeneca used sulfonyl fluoride warhead to rationally design covalent fragments targeting Tyr101 of antiapoptotic protein Bcl-xl.^107^ Subsequent structure-based elaboration led to more potent compound with time-dependent biochemical potency against Bcl-2 family proteins-BIM/BAK interactions. These are important examples of how covalent fragment screens combined with chemistry optimization could generate novel chemical tools to target challenging PPIs. Over the coming decade, we expect to see fragment-based covalent ligand discovery to rise to challenges posed by PPIs.

### Covalent fragments for allosteric regulator development

Although covalent allosteric inhibitors have recently gained interest in drug discovery for challenging target proteins,^108,109^ the systematic discovery of those hidden low-occupancy allosteric sites remains highly challenging. Due to their improved ability to access cryptic sites, fragments could be used for discovery of allosteric binders as well as identification of functionally relevant secondary binding sites on proteins.^16^ In the context of covalent fragments, Keedy *et al.* screened a disulfide-capped fragment library containing around 1600 fragments against the engineered cysteine mutant (Lys197Cys) of tyrosine phosphatase 1B (PTP1B), a target that has been difficult to selectively inhibit *via* active site directed route.^110^ After a combined labeling study and a follow-up optimization, fragment 2 was identified as a noncompetitive allosteric inhibitor in an enzymatic assay. Crystallographic studies clearly demonstrated that fragment 2 was covalently tethered to Lys197Cys instead of the catalytic cysteine in the active site, with protein conformational changes similar to those induced by endogenous regulatory mechanism. Additionally, Keedy *et al.* identified additional fragments that were found to bind to the same location in wild type (WT) PTP1B, suggesting that identified site could be exploited for allosteric inhibitor development.^110^ Overall, this work highlights the utility of covalent fragments for allosteric site binder discovery.

### Development of proteolysis targeting chimeras (PROTACs)

Another compelling application is the use of covalent fragments to develop new modalities, such as proteolysis targeting chimeras (PROTACs). Recently, PROTACs emerged as an appealing strategy in drug discovery. These heterobifunctional small molecules recruit E3 ligases to the target of interest, subsequently resulting in target ubiquitination and degradation.^111^ Although there are more than 600 putative E3 ligases available in human proteome, only a handful of them (CRBN, VHL, MDM2 and cIAP1) has been successfully employed to develop degraders due to the lack of well-characterized E3 ligase ligands.^112^ Thus, identifying additional E3 recruiters by covalent fragment screen may offer great opportunity for the development of novel electrophilic PROTACs with distinct pharmacological profiles. For example, “scout fragments” with chloroacetamide or acrylamide warheads have recently been coupled to selective ligands to screen bifunctional covalent degrader molecules.^113^ This effort led to the discovery of nuclear-localized FKBP12 degrader KB02-SLF and BRD4 degrader KB02-JQ1, which hijacked E3 ligase DCAF16 for nuclear protein degradation. This is an exciting example that demonstrated the feasibility of chemoproteomics-based covalent fragment screen in electrophilic PROTACs development.

Additionally, a recent ABPP-based covalent screen against purified RNF4, a RING-domain E3 ligase identified new covalent fragments that target this protein.^114,115^ The most promising hit TRH 1-23 was shown to label either Cys132 or Cys135, two cysteine residues that form one of zinc-coordinating sites within the RING domain. Although disrupting either one of these sites through mutagenesis was previously shown to inhibit RNF4 function, Ward *et al.* included evidence to suggest that RNF4 retains *in vitro* self-ubiquitination activity, suggesting that TRH 1-23 did not disrupt RNF4 E3 ligase function. Further work is needed to characterize this biding and explain the lack of impact on E3 ligase activity, despite the targeting of the two cysteines essential for zinc binding. The same study also demonstrated that these hits can be used to generate PROTACs featuring covalent E3 recruiting arm. The PROTACs were validated based on targeted protein degradation of BRD4.

Overall, these examples highlight the potential utility of covalent fragments to discover “hidden” druggable sites and serve as a starting point for developing ligands, including PROTACs. Although covalent PROTACs have initially been somewhat controversial, there is now broader acceptance that PROTACs featuring a covalent handle on E3 ligase recruiting arm may have benefits. Very recently, Bond *et al.* reported an effective degrader of Kras-G12C by coupling a covalent Kras-G12C ligand with a VHL E3 recruiter. While there have been very few examples of using covalent target ligands for bifunctional degraders, going forward it would be interesting to realize utility of covalent ligands to degrade high-value targets that have been recalcitrant to ligand discovery.

## Outlook and future directions

Covalent fragment-based ligand discovery has already yielded a significant number of useful and synthetically accessible covalent fragments for drug discovery. These binders provide a solid starting point for developing probes for the ligandable hot spot of the protein targets. However, transforming a covalent fragment hit into an optimized molecule still requires a considerable medicinal chemistry effort. Here, strategies such as structure-guided drug design, covalent SAR (covSAR) interpretation,^116^ fragment merging^117^ and similar can help speed up the process and we expect that these efforts will continue to be merged into unified campaigns towards novel leads and drugs.

Chemoproteomic methods for characterizing covalent fragments can play an important role towards the identification of diverse electrophiles with suitable properties for future covalent fragment library expansions. For instance, the incorporation of masked thiol-reactive fragments into commonly used cysteine-directing library that could be triggered under precisely defined physiological conditions has recently been shown as a viable option to diversify the cysteine-directing library.^118,119^ We expect that further expansion of the covalent fragment chemical space will occur as the efforts move towards targeting alternative residues beyond cysteines, as we discussed above. Projecting forward, the identification of covalent fragments modifying those protein nucleophiles (lysine, serine, histidine, tyrosine, methionine, glutamic acid *etc.*) would help expand the scope of covalent fragment and covalent inhibitor use, as well as the target space for small molecule ligand discovery and development. We also expect that the growing amount of chemoproteomic data will further feed into development of new computational methods, including machine learning algorithms for virtual screening of covalent fragments for a target of interest.

One of the main concerns related to the use of covalent fragments is their potential promiscuous nature, which may not directly correlate with intrinsic reactivity. In this context, frequent hitters, such as aminothiazole chloroacetamides that are not significantly more reactive on average, have been recognized as promiscuous binders.^38^ However, we should still be careful about covalent warheads with high reactivity (*k* > 1 × 10^−7^ M^−1^ s^−1^) with the caveat about their potential non-specific cell toxicity. Some compounds with non-specific reactivity, such as quinones, maleimides and *N*-hydroxysuccinimide (NHS)-esters, have been flagged as pan-assay interference compounds (PAINS)^120,121^ and should also be avoided in the construction of covalent fragment library.

We believe that in the foreseeable future, the covalent fragments will continue to help us assess biologically relevant chemical space and systematically identify more chemical-tractable targets in the native biological systems. Additionally, covalent fragments and the chemical probes and lead compounds they spawn will be of essential importance for facilitating broader study of biological processes and how they contribute to normal human physiology as well as disease. In summary, we expect that covalent fragments will be of growing interest to chemical biologist, as well as those interested in drug discovery and development.

The fact that covalent fragment discovery has already succeeded in enabling actual drug candidates against KRAS, the notorious and most sought-after target in oncology also leads to a big question for the field. Was this a lucky, one-off case where covalent targeting just happened to match this particular challenging field or is this a preview, of continued and increased impacts that these approaches will have on drug discovery as the improved methods, larger libraries, and increased focus start to bear fruit? We eagerly await further forthcoming efforts in this field and hope we are truly entering a new era for targeted therapy.

## Conflicts of interest

N. S. G. is a founder, science advisory board member (SAB) and equity holder in Gatekeeper, Syros, Inception, Jengu, C4, B2S, Aduro and Soltego. The Gray lab receives or has received research funding from Novartis, Takeda, Astellas, Taiho, Janssen, Kinogen, Voronoi, Her2llc, Epiphanes, Deerfield and Sanofi. L. H. J. receives research funding from Deerfield. M. K. is a paid consulting editor at Life Science Editors. M. P. P. is an employee and have equity in Vividion Therapeutics. J. C. is a consultant for Jengu, Allorion, and Soltego, and an equity holder in Allorion and M3 bioinformatics & technology.

## Supplementary Material
