# Correction: Fragment-based covalent ligand discovery

**DOI:** 10.1039/d1cb90008k

**Published:** 2021-02-22

**Authors:** Wenchao Lu, Milka Kostic, Tinghu Zhang, Jianwei Che, Matthew P. Patricelli, Lyn H. Jones, Edward T. Chouchani, Nathanael S. Gray

**Affiliations:** Department of Cancer Biology, Dana-Farber Cancer Institute Boston MA 02215 USA nathanael_gray@dfci.harvard.edu; Department of Biological Chemistry and Molecular Pharmacology, Harvard Medical School Boston MA 02215 USA; Center for Protein Degradation, Dana-Farber Cancer Institute Boston MA 02215 USA; Vividion Therapeutics La Jolla CA 92121 USA; Department of Cell Biology, Harvard Medical School Boston MA 02215 USA

## Abstract

Correction for ‘Fragment-based covalent ligand discovery’ by Wenchao Lu *et al.*, *RSC Chem. Biol.*, 2021, DOI: 10.1039/d0cb00222d.

The authors regret that an incorrect version of Fig. 2 was included in the original article, where the structure of Sulfopin in Fig. 2D was incorrectly shown. The correct version of Fig. 2 is presented below.

**Fig. 2:**
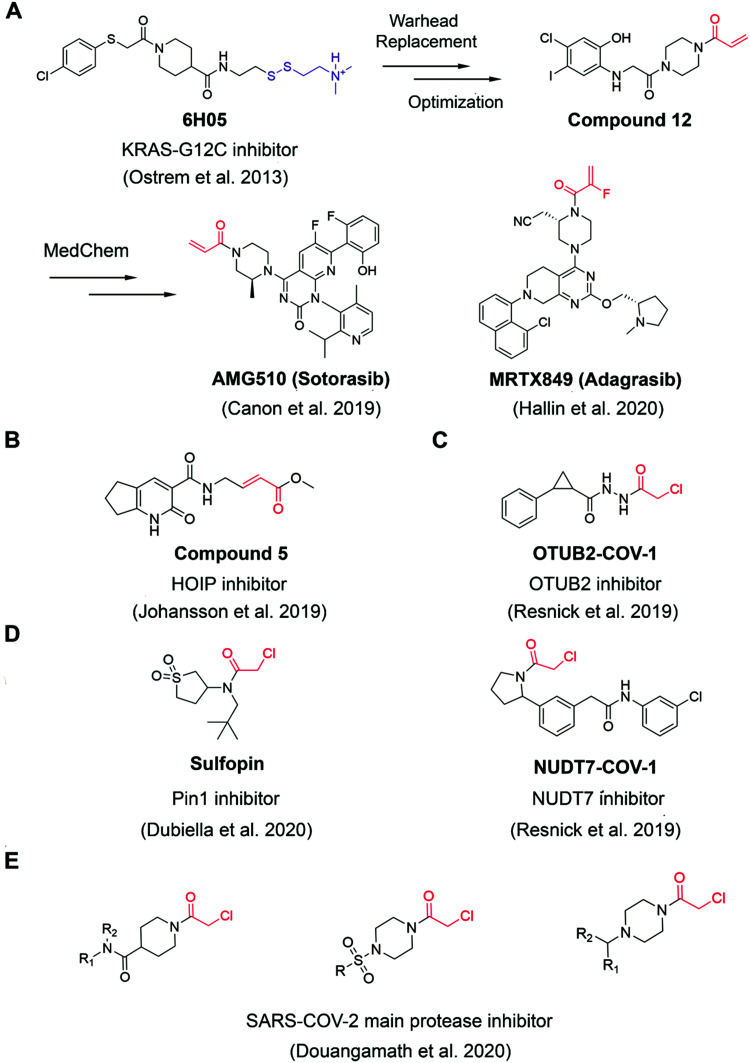
The structures of representative well-characterized electrophilic fragments identified from target-based screening strategies in recent years. (A) KRAS-G12C allele-specific covalent fragment (6H05) identified from tethering screen, which was further elaborated to compound 12.^31^ This inspired numerous groups to develop further optimized inhibitors, within which AMG510^33^ and MRTX849^36^ successfully entered clinical trials. (B) Compound 5 targets the active cysteine (C885) of HOIP.^37^ (C) OTUB2-COV-1 targets the active cysteine (C51) of OTUB2 and NUDT7-COV-1 target C73 of NUDT7.^38^ (D) Sulfopin targets the active cysteine of Pin1 (C113).^39^ (E) Representative covalent fragment scaffolds target the active cysteine (C145) of SARS-COV-2 main protease (Mpro).^40^

The Royal Society of Chemistry apologises for these errors and any consequent inconvenience to authors and readers.

## Supplementary Material

